# Supplementary material for: Does grit protect against the adverse effects of depression on academic achievement?
Source: PLoS One. 2023 Jul 7;18(7):e0288270. doi: 10.1371/journal.pone.0288270 (PMC10328250; doi:10.1371/journal.pone.0288270)

**Supplemental Materials for Does Grit Protect Against the Adverse Effects of Depression on Academic Achievement?**

**Research questions and hypotheses**

We also hypothesized that:

(1) depressive symptoms will be negatively related to GPA, as found in previous research [4],

(2) depressive symptoms would be negatively related to grit, as found in previous

research [7],

(3) grit would be positively related to GPA, as found in previous research [7], and

(4) grit would act as a buffer on the relationship between depressive symptoms and GPA, such that the relationship between depressive symptoms and GPA would be less negative when self-reported grit is high [4,6].

Duckworth [6] suggests that correlations between grit and achievement are stronger when social desirability is accounted for; however, Credé [7] suggests that this relationship may be overestimated due to social desirability. Thus, we also wanted to explore how social desirability affects the relationship between grit and GPA when depressive symptoms are not included in the relationship. We thus also evaluated the following competing hypotheses that either (a) grit’s relationship with GPA would be more positive when social desirability is high [6], or (b) grit’s relationship with GPA would be less positive when social desirability is low [7].

**Results**

**Data analytic plan**

Initial correlational analyses were run to assess the relationship between (1) depressive symptoms and grit, (2) depressive symptoms and GPA, and (3) grit and GPA. All other hypotheses were examined using the PROCESS macro for SPSS (Hayes, 2012, 2013). Three moderation hypotheses were examined using PROCESS Model 1 to assess whether (1) grit moderated the relationship between depressive symptoms and GPA, (2) social desirability moderated the relationship between grit and GPA, and (3) social desirability moderated the relationship between depressive symptoms and GPA.

**Correlational analyses**

Correlational analyses were conducted to examine initial relationships between variables. Depressive symptoms were negatively related to grit *r*(518) = -.40, *p* < .001, providing support for our first hypothesis. Depressive symptoms were negatively related to GPA, *r*(518) = -.18, *p* < .001, providing support for our second hypothesis. Grit was modestly and positively related to GPA *r*(518) = .12, *p* < .01, providing support for our third hypothesis and consistent with previous literature, in which effect sizes range from .06 to .25 (Duckworth & Gross, 2014; Duckworth et al., 2007).

**Depressive symptoms, grit, and GPA**

To investigate our fourth hypothesis, we conducted a moderation analysis to examine whether grit moderated the relationship between depressive symptoms and GPA and further explore how the presence of grit may protect against the previously established negative relationship between depressive symptoms and GPA (Bruffaerts et al., 2018; Deroma et al., 2009; Eisenberg et al., 2009; Hysenbegasi et al., 2005). In line with previous research, we hypothesized that grit would moderate the relationship between depressive symptoms and GPA, such that the relationship between depressive symptoms and GPA will be less negative when self-reported grit is high (Duckworth et al., 2007; Hysenbegasi et al., 2005). Our results do not support our fourth hypothesis and suggest that grit does not buffer against the negative effects of depressive symptoms on GPA; rather, the relationship between depressive symptoms and GPA is maintained even when grit is included in the model. See Figure S1 for these results. The main effect of depressive symptoms, *b* = -.15, 95% CI [-.240, -.051], *t* = -3.03, *p* = .003, was significant, evidencing a negative relationship with GPA; however, grit was not uniquely predictive of GPA, *b* = .05, 95% CI [-.038, .145], *t* = 1.14, *p* = .25. Additionally, the interaction was not significant, *b* = .04, 95% CI [-.036, .125], *t* = 1.14, *p* = .28.

**Figure S1. Relationship Between Depressive Symptoms and GPA at Levels of Grit**


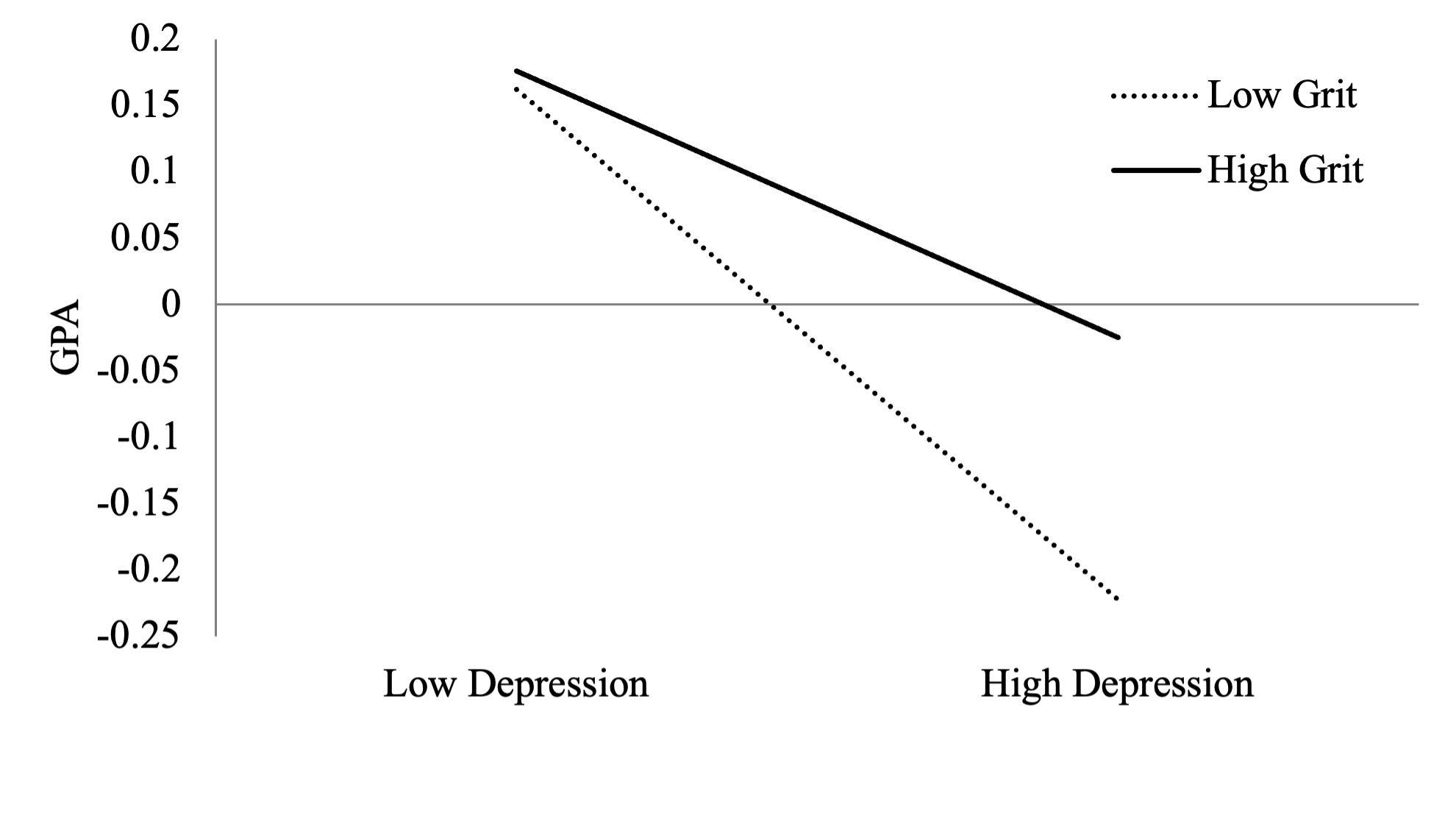


**Depressive symptoms, social desirability, and GPA**

To investigate our competing hypothesis, we conducted a moderation analysis to examine the interaction between depressive symptoms and social desirability in predicting GPA. Our results did not support our hypothesis as social desirability did not affect the relationship between depressive symptoms and GPA. See Fig S2 for these results. The main effect of depressive symptoms remained significant, *b* = -.20, 95% CI [-.282, -.112], *t* = -4.53, *p* < .001, evidencing a negative relationship with GPA, but the main effect of social desirability, *b* = -.08, 95% CI [-.170, .001], *t* = -1.94, *p* = .053, and the interaction between depression and social desirability, *b* = .05, 95% CI [-.037, .132], *t* = 1.10, *p* = .27, were non-significant.

**Fig S2**. **Relationship Between Depressive Symptoms and GPA at Levels of Social Desirability (MC)**


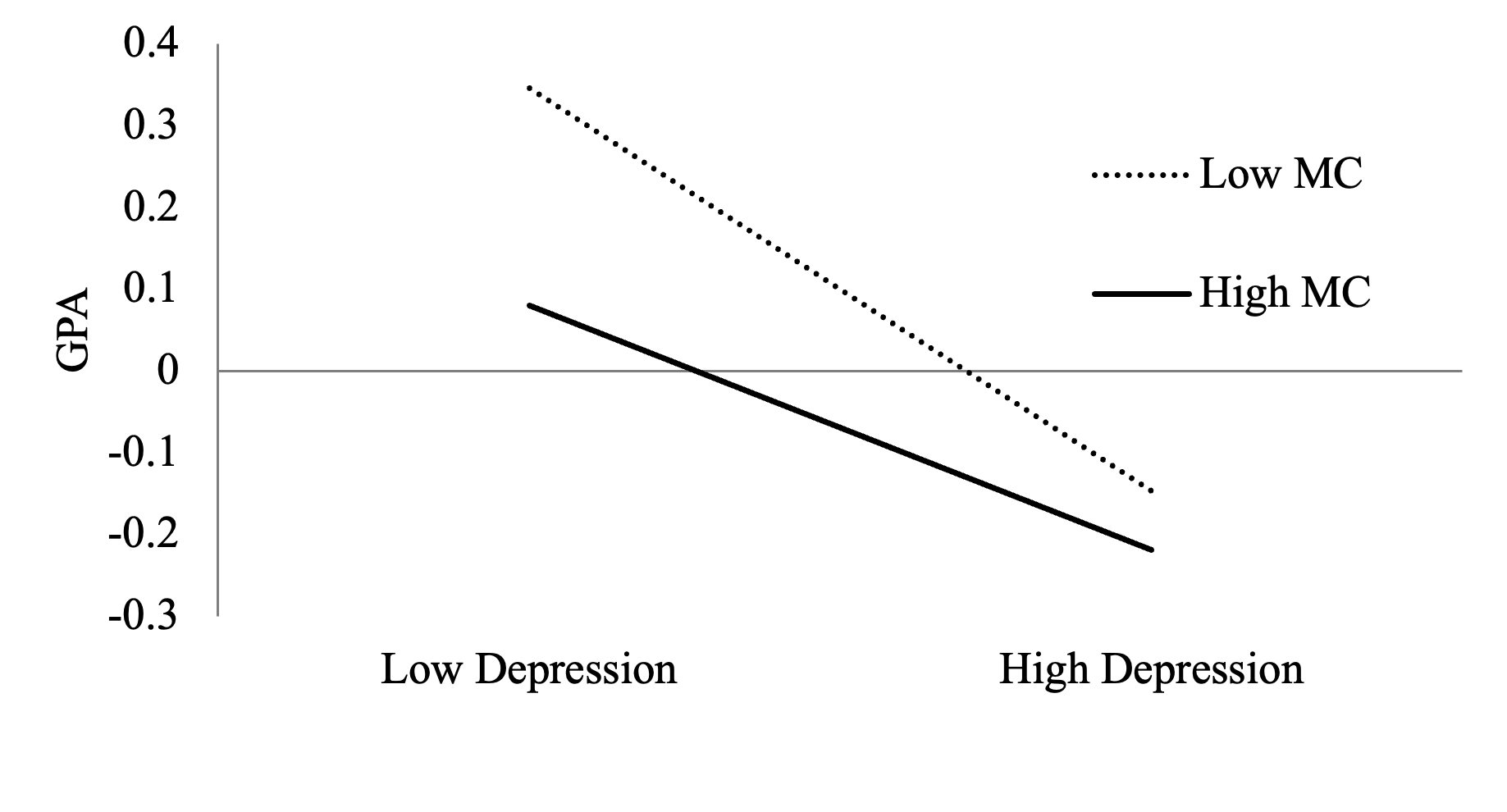


**Social desirability, grit, and GPA**

To explore competing hypotheses about how social desirability affects the relationship between grit and GPA, the second moderation analysis assessed whether social desirability moderated the relationship between grit and GPA (Chang, 2014; Credé et al., 2016; Duckworth et al., 2007; Duckworth & Quinn, 2009). Grit, *b* = .16, 95% CI [.067, .248], *t* = 3.43, *p* < .001, and social desirability, *b* = -.10, 95% CI [-.189, -.009], *t* = -2.16, *p* = .031, were each uniquely predictive of GPA, such that higher grit and lower social desirability were associated with higher GPA. When examining the interaction, social desirability did not significantly moderate the relationship between grit and GPA, *b* = -.07, 95% CI [-.148, .007], *t* = -1.78, *p* = .076. We nevertheless probed the interaction (both due to theoretical interest and as is automatic in the PROCESS module). At low levels of social desirability, grit remained positively associated with GPA, *b* = .23, 95% CI [.106, .350], *t* = 3.67, *p* <.001. However, at high levels of social desirability, the relationship between grit and GPA was no longer significant, *b* = .09, 95% CI [-.028, .204], *t* = 1.48, *p* = .14. Although non-significant, these results, as seen in Fig S3, suggest that grit may be predictive of GPA when social desirability is low.

**Fig S3. Relationship Between Grit and GPA at Levels of Social Desirability (MC)**


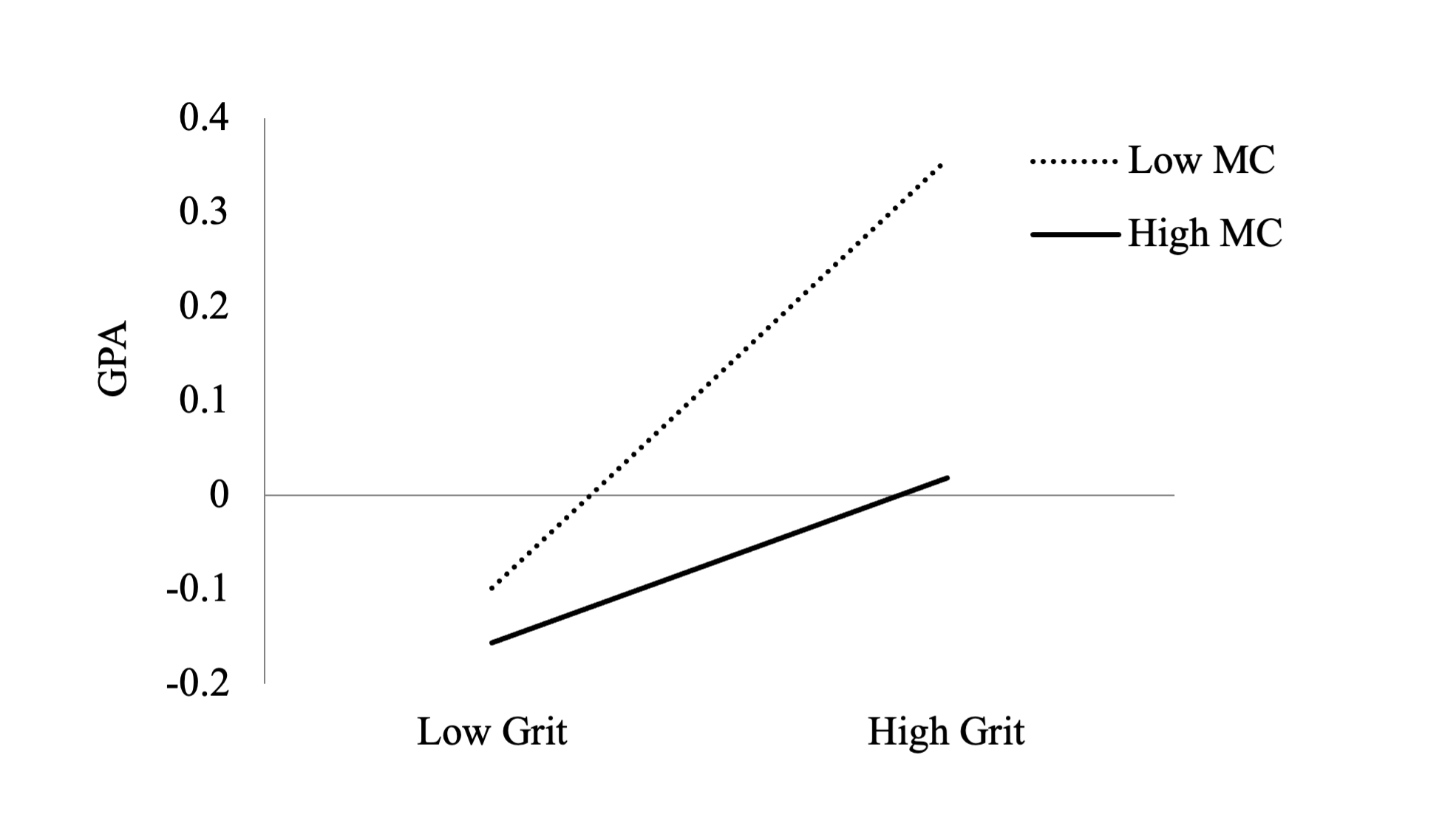

Supplement: S1 File — The supporting information contains our other a priori hypotheses, data analytic plan, and results. (DOCX) [file pone.0288270.s001.docx]
