# Supplementary material for: Does grit protect against the adverse effects of depression on academic achievement?
Source: PLoS One. 2023 Jul 7;18(7):e0288270. doi: 10.1371/journal.pone.0288270 (PMC10328250; doi:10.1371/journal.pone.0288270)
Supplement: Attachment — Submitted filename: Responses to Reviewers.docx [file pone.0288270.s003.docx]

**Reviewers' comments:

Reviewer's Responses to Question

Please use the space provided to explain your answers to the questions above. You may also include additional comments for the author, including concerns about dual publication, research ethics, or publication ethics. (Please upload your review as an attachment if it exceeds 20,000 characters)**

**Reviewer #1: Thank you for the significant revisions to the paper. I think it definitely improved compared to the previous version. This being said, there are some aspects that I think could still be clarified to improve the value of the paper.

Abstract
I think the abstract is now much easier to follow. However, I also think that the role of social desirability in the model is still somewhat unclear and unexpected given that the very first sentences (lines 27-28) mainly refer to grit. I would suggest the authors describe the inclusion of social desirability right after grit so that it is clear how/why this is part of the study.**

Thank you for taking the time to review our manuscript again and provide this feedback.

We have added information about social desirability in the abstract. **Intro:
I find this version of the introduction much more compelling and better structured.
I still have the following comments:
Lines 72-73: I do not follow how the fact that grit may not consistently predict academic success across domains is linked to your study. This seems to suggest that you investigate this link across different domains, but this was not the case.**

We have edited the language in this paragraph to better indicate that grit and academic

performance may not be as closely related as prior research has suggested.

**I think that the opening of the paragraph “Research questions and hypotheses” could be clearer. On line 103 you report that “individuals with higher levels of grit experience lower depressive symptoms”, implying that (high) grit leads to (lower) depressive symptoms. Please report a reference here so that it is clear upfront that this is what previous research shows – and not what your study is about.**

We have added the relevant references on pg 6.

**Results:
When reading your results, I was wondering whether you controlled for age and year of enrollment. I would be curious to know if anything changes.**

Thank you for this suggestion. We have conducted the main analyses with class year and age as covariates, which were both significant effects; however, including them in the moderated moderation did not change the overall findings. We included this information in footnote 3 on pg 12.

**Line 246: You report that “grit is not a promising factor… when considering social desirability”. I would suggest adding - “even” when considering social desirability-, given that your results show that grit alone was also not significantly related to achievement.**

We have added this to the sentence.

**Line 247-248: How did your results show that social desirability impacts the validity of grit when assessed via self-reported measures?**

Thank you for this clarification. We have added the following language on pgs 14-15 to clarify this implication:

“Moreover, our results in the Supplemental Materials provide further insight into the relationship between grit and GPA. Specifically, when examining whether social desirability moderated the relationship between grit and GPA, we found main effects for both grit and social desirability on GPA. However, we probed the moderation and found that grit was only significantly related to GPA at low levels of social desirability (and not high levels of social desirability), thus supporting prior research indicating that social desirability may impact the validity of grit when assessed via self-report measures [7].”

**Minor:
Given that your study is cross-sectional, please avoid the use of terms that imply causality (e.g., impact, line 35).**

We have removed any causal language when discussing our findings.

**Reviewer #3: The efforts put in by the authors are commendable, considering the criticality of the opted research topic. Moreover, the research team has highlighted the academic and practical implication of the presented study very well. Having that said, the following suggestions will assist to improve the manuscript further:

1- Introduction & Literature Support: The authors have successfully communicated the need of conducting the research on the topic of interest, but the provided literature support (in-depth explanation with enough references) for the variables of interest and hypothesized relationships (direct & indirect) is missing. Therefore, a potential reader my remain unconvinced to see the broader impact of the selected variables.**

We thank the Reviewer for taking the time to review our manuscript again and for providing this feedback on our Introduction. We have added additional information and references in the Introduction to provide more support for our hypotheses. There are now 28 relevant references in advance of the introduction of our hypotheses.

**2- Method & Analysis: The presented research does a good job in explaining the selected sample and concluding the results. Having that said there are few gaps that need to be addressed including:
- Why was the initial sample of 625 (final count=520) was opted? An explanation is required in terms of relating it to the population of interest. As this will define the scope of generalization for the presented research.**

Thank you for asking for this clarification. We have added a footnote on pg 7 to indicate our reasoning for collecting 625 participants initially.

**- Why was G-Power utilized in comparison to the other statistical tools to determine the results. An brief explanation supporting the accuracy of the opted tool along with its capability to evaluate large sample size and complex structures will suffice.**

We have added a brief description of our rationale for using G*Power on pg 12.

**- For readers there is very less visibility of the detailed measurement model results (instrument's reliability, validity, correlation, coefficient of determination, multi-collinearity and more). The less evidence of the said results and associated discussion reduces the support for the structural model results of the current research.**

Thank you for pointing this out. We have added additional information to describe the self-report measures used in our analyses. We have also added more descriptive information in Table 1. At the request of a reviewer in a previous review, we moved our additional analyses, including correlations, to the supplemental materials.

**- Overall, material and method sections need to be revised for their comprehension and filling in the above mentioned gaps. This will result significantly increased credibility of the current research.**

Thank you again for your feedback for our Material and Method sections. We believe that our revisions improve comprehension and credibility of the current study.

**Discussion: Expansion of discussion in terms of theoretical and practical implications of the present research needs to be done.**

Thank you for this suggestion. We have added an additional paragraph on pgs 13-14 to expand on our discussion and the current study’s implications. We have also revised the Conclusion to succinctly note the theoretical and practical implications of the present research.
